# Supplementary material for: Normalization governs attentional modulation within human visual cortex
Source: Nat Commun. 2019 Dec 11;10:5660. doi: 10.1038/s41467-019-13597-1 (PMC6906520; doi:10.1038/s41467-019-13597-1)
Supplement: Supplementary file 1 — Supplementary Information [file 41467_2019_13597_MOESM1_ESM.pdf]

Normalization governs attentional modulation within human visual cortex

Ilona M. Bloem[1,2*] & Sam Ling[1,2]

[1] Department of Psychological and Brain Sciences, Boston University, Boston

[2] Center for Systems Neuroscience, Boston University, Boston

Supplementary Information, including:

Number of Supplemental Figures: 9

References

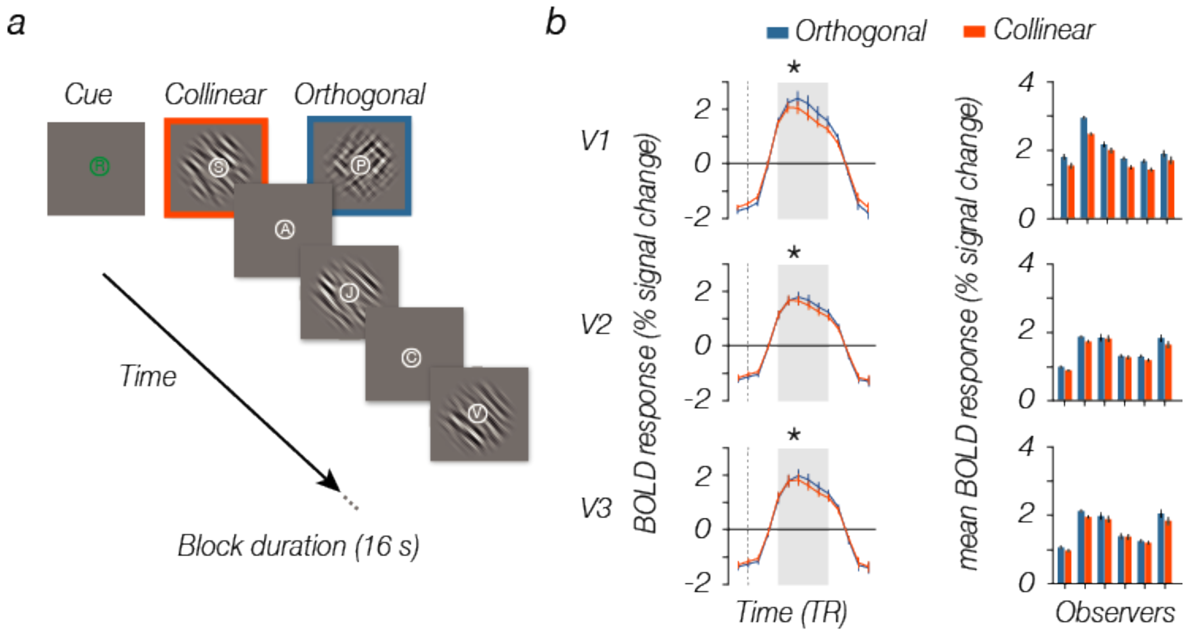

Supplementary Figure 1. Measuring tuned normalization. **a.** Schematic of an example block sequence. Either collinear (45°/45° or 135°/135°) or orthogonal (45°/135° or 135°/45°) stimuli were presented during a 16 sec block (2s cue period, followed by stimuli presented for 250ms on, 250ms off), while participants performed a fixation task, discriminating target letters in a rapid letter stream. **b.** Mean BOLD responses (left panels) were larger for orthogonal compared to collinear stimuli configurations. Grey highlighted part of the BOLD response reflects the section that contributed to the average BOLD response for each participant (right panels). Stimuli are modified for illustrative purposes; N = 6; error bars denote ±1 S.E.M..

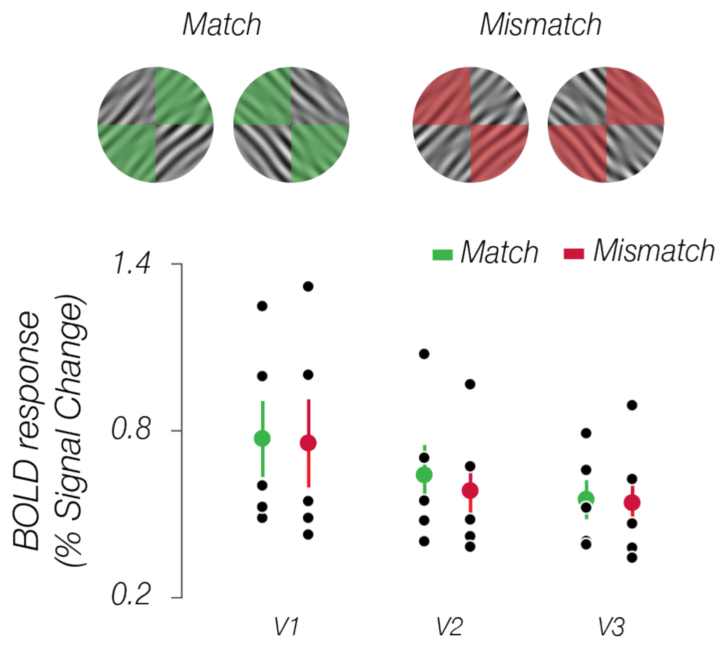

Supplementary Figure 2. Measuring radial bias. Population receptive field information was used to break up responses within each region based on their retinotopic preference, and subdivided into one of two categories, depending on spatial preference and the orientation that was presented. In the Match configuration, the orientation of a presented stimulus matched that of the preferred retinotopic radial axis, whereas in the Mismatch condition, it did not. Our data did not show a significant radial bias, as the mean BOLD responses did not differ between Match and Mismatch conditions (repeated measures ANOVA, interaction effect, and main effects between radial axis and visual area were not significant p>0.05); N=5, error bars denote ±1 S.E.M..

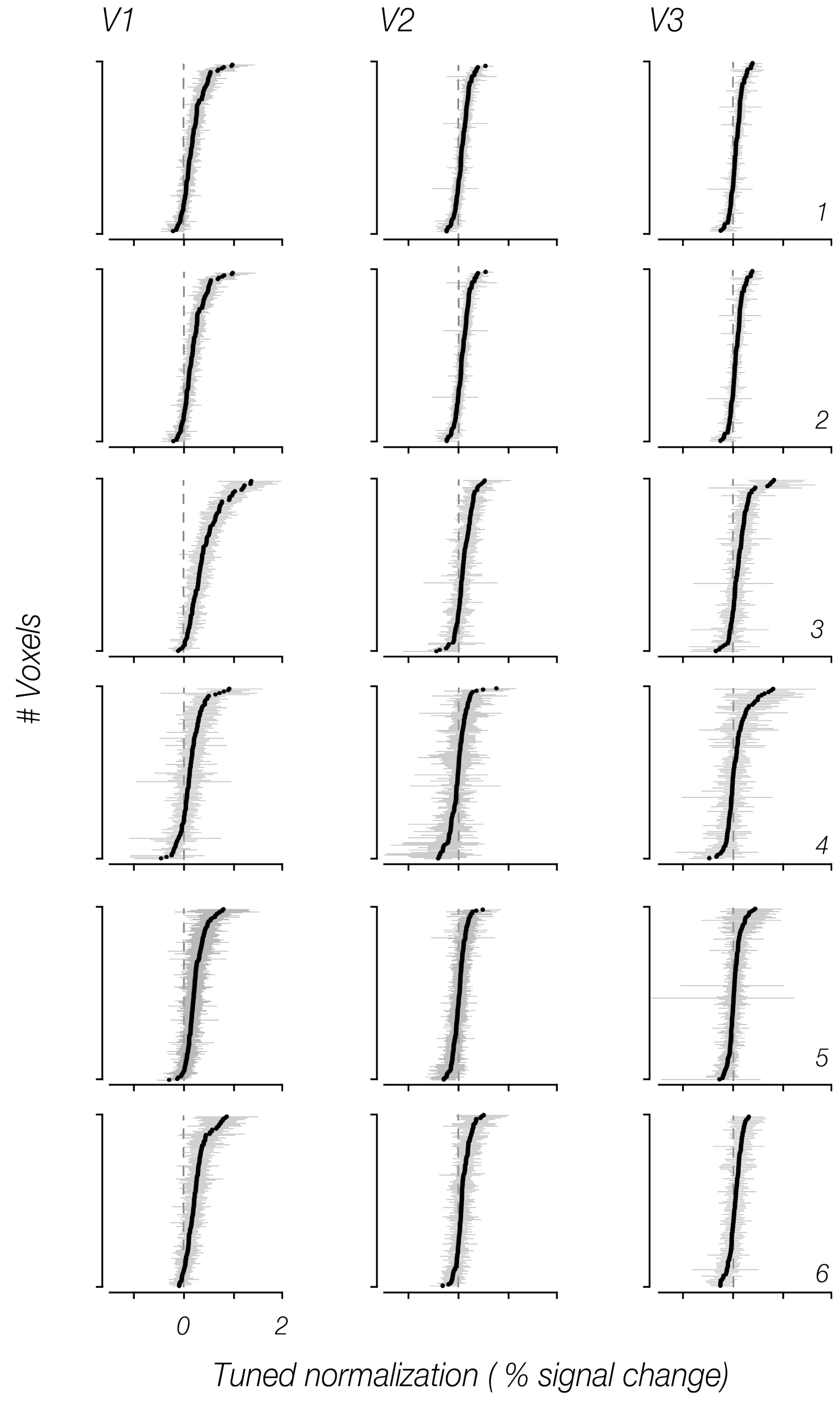

Supplementary Figure 3. Individual participant data illustrating the reliability of the tuned normalization measure. We computed the bootstrapped 95% confidence interval (1000 repetitions) for the top 25% voxels within each region of interest for each observer. Black dots illustrate the average mean tuned normalization strength for each voxel, while the grey lines denote the confidence interval. Voxels are ranked according to their respective tuned normalization strength. This reveals that the voxel-wise measure of normalization is quite stable; N = 6.

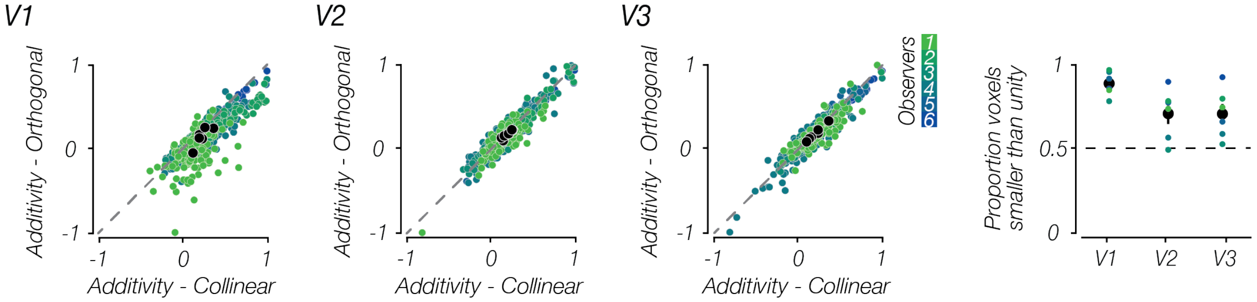

Supplementary Figure 4. Voxel-wise relationship of the deviation from additivity for both stimuli configurations in V1-V3. BOLD responses were normalized for each participant (N=6). The average response for each participant demonstrated robust feature-tuned normalization, whereby stimuli comprised of collinear orientations were more sub-additive, and thus more strongly normalized, than stimuli that contained orthogonal orientations (repeated measures ANOVA interaction effect $F(2,15) = 7.85$, $p = 0.005$, $\eta_p^2 = 0.511$; tuned normalization post-hoc analysis two-sided paired t-test; V1: $t(5) = 6.00$, $p = 0.0057$, $d = 2.44$, V2: $t(5) = 3.82$, $p = 0.0370$, $d = 1.56$, and V3: $t(5) = 3.44$, $p = 0.0551$, $d = 1.42$, Bonferroni corrected). Individual voxel responses reveal large heterogeneity in the magnitude of sub-additivity within a region. Comparing the difference between the hypothetical additive sum with both collinear and orthogonal stimulus configurations revealed a consistent pattern, with the collinear configuration exhibiting larger sub-additivity compared to the orthogonal configuration – an effect that decreases in magnitude along the visual hierarchy. Small dots indicate individual voxels for each observer; larger black dots represent the whole ROI average per observer. Right panel indicates the proportion of voxels that falls below the unity line and thus is demonstrate tuned normalization. Large black dots indicate group average; smaller colored dots represent individual observers; error bars denote ±1 S.E.M..

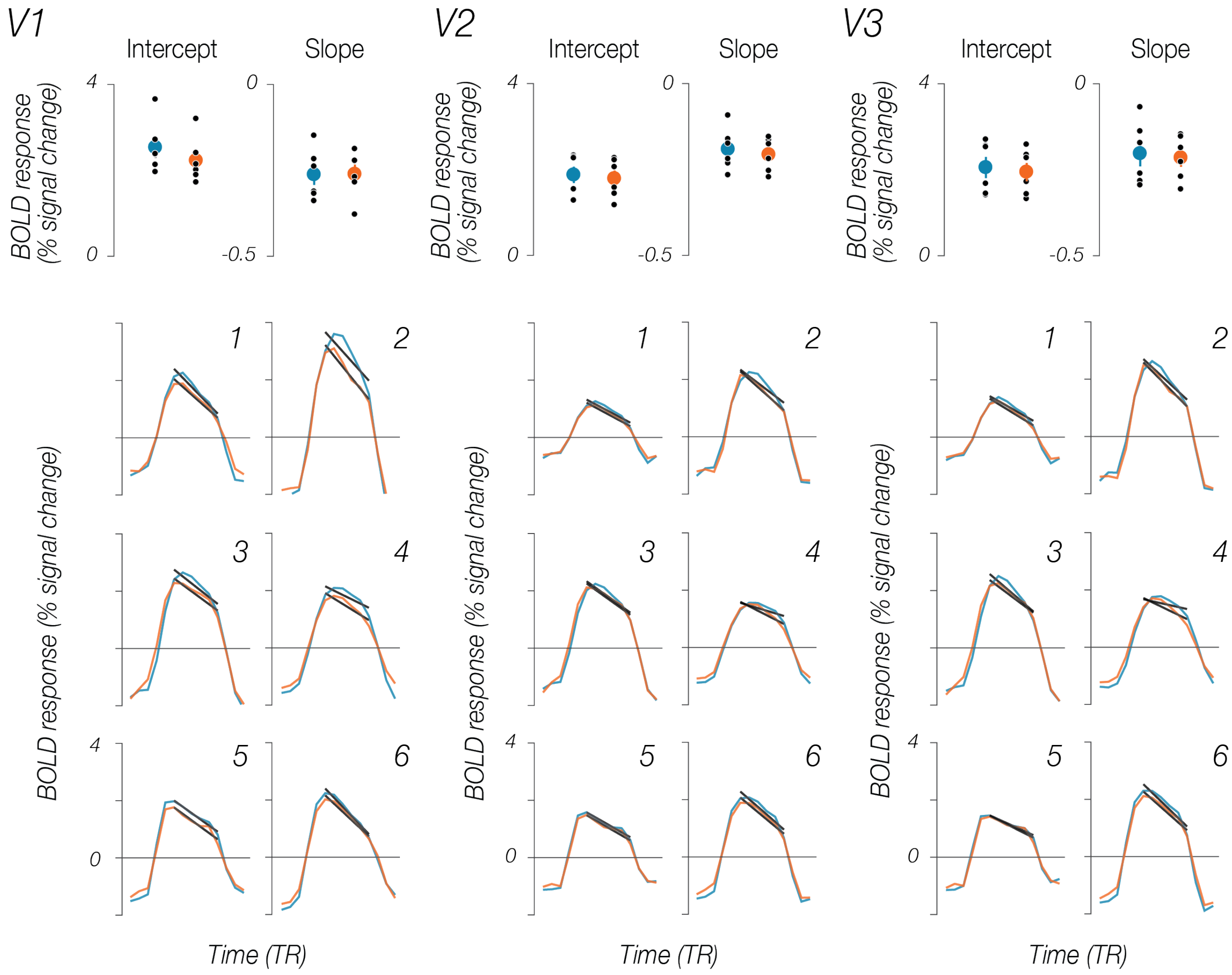

Supplementary Figure 5. Differences between stimuli configurations are not explained solely by adaptation. Evoked responses (averaged across blocks, excluding the first 2 TRs to account for the hemodynamic lag) in both stimuli configurations were fitted independently for each observer with a linear model. While the intercept of this model differed between Orthogonal (blue) and Collinear (orange), reflecting an overall mean BOLD difference (repeated measures ANOVA interaction effect $F(2,15) = 10.00$, $p = 0.002$, $\eta_p{}^2 = 0.572$; post-hoc analysis two-sided paired t-test; V1: $t(5) = 8.67$, $p = 0.0010$, $d = 3.53$, V2: $t(5) = 2.70$, $p = 0.1278$, $d = 1.10$, and V3: $t(5) = 2.30$, $p = 0.2097$, $d = 0.95$, Bonferroni corrected), there was no difference in the estimated slope between the two conditions, suggesting that adaptation did not play a major role in the difference between the two stimuli configurations (repeated measures ANOVA: interaction effect, and main effects between stimulus configuration and visual area were not significant $p>0.2$); N = 6; error bars denote ±1 S.E.M..

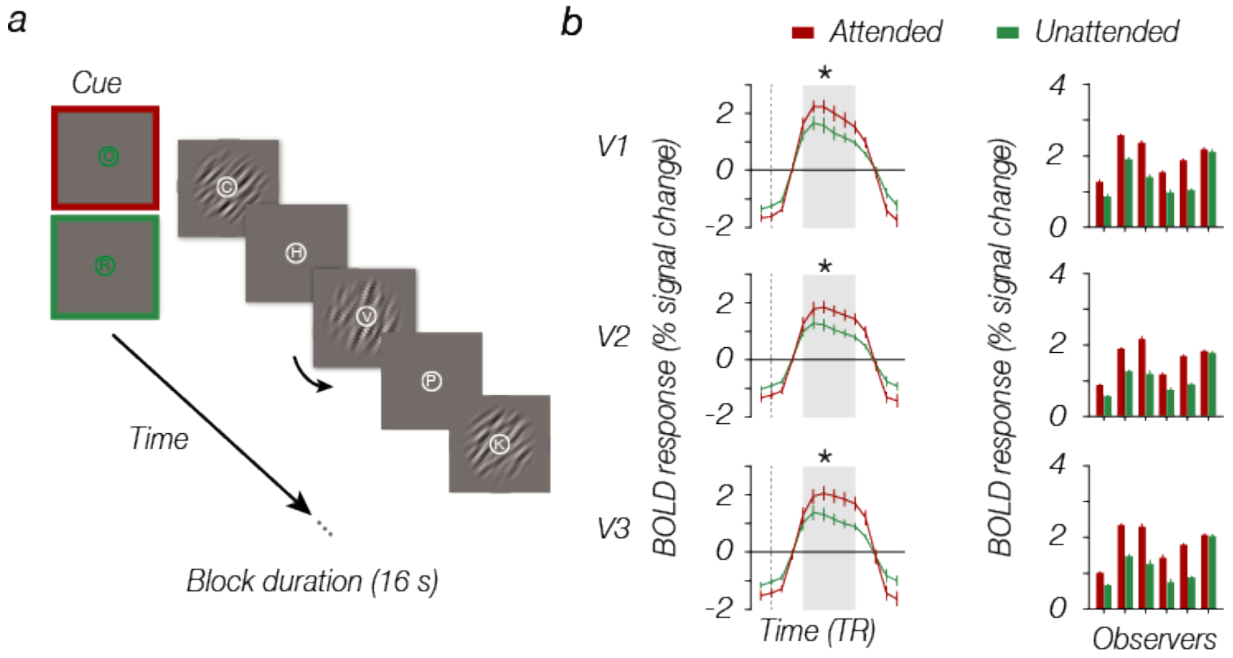

Supplementary Figure 6. Measuring attention modulation. **a.** Schematic of an example block sequence. A brief cue (2 sec) instructed observers to either attend towards the grating (fine orientation discrimination task), or attend away from the grating (RSVP task at fixation). Both orientation and letter targets would appear throughout a block, only the initial cue informed the participant which task to perform. **b.** Attending towards the stimulus resulted in a larger BOLD response compared to the unattended condition (left panels). Grey highlighted part reflects the section that contributed to the average BOLD response for each participant (right panels). Stimuli are modified for illustrative purposes; N = 6; error bars denote ±1 S.E.M..

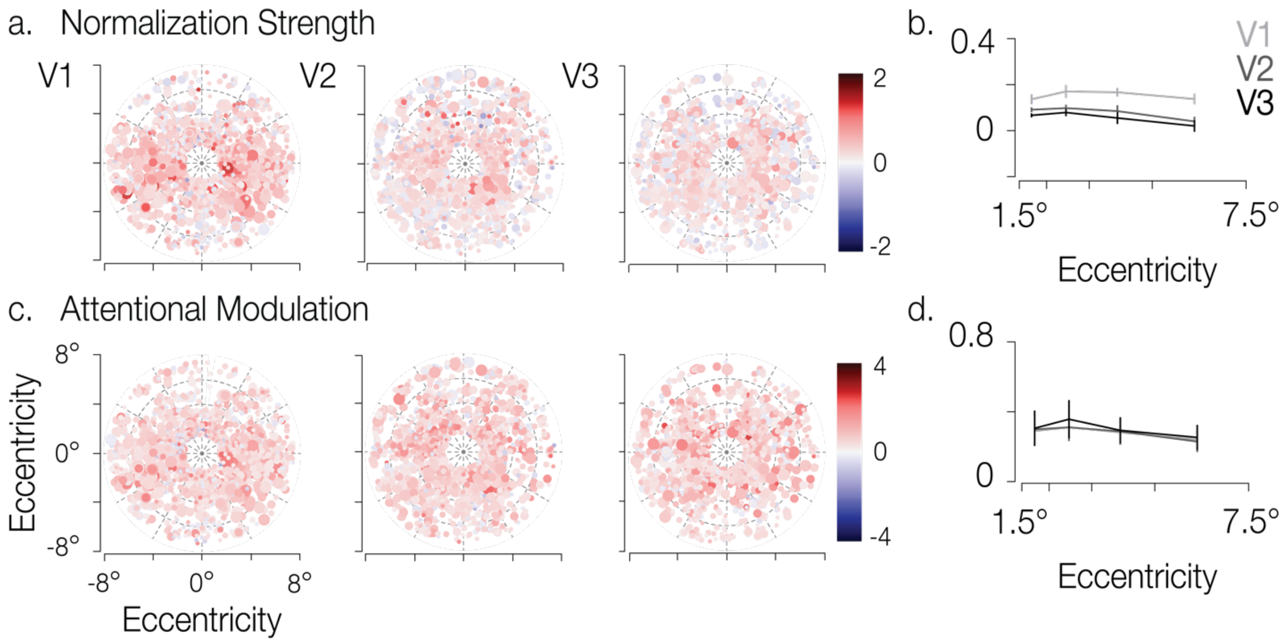

Supplementary Figure 7. Retinotopic preference for tuned normalization and attentional modulation. **a.** Tuned normalization strength across spatial position for V1-V3, combined over observers (N=5; as one observer did not complete pRF scanning). Each dot represents a voxel's retinotopic preference, and its size indicates the goodness of fit from an independent localizer. **c.** Attentional modulation across spatial position. **b-d.** Both tuned normalization and attention measures as a function of eccentricity. Both indices were allotted into 4 logarithmically spaced bins, based on eccentricity (range between 1.5°-7.5°; collapsed across angular preference). To estimate potential biases in both measures of interest, based on retinotopic preference, a linear model was fitted to each measure and region of interest, revealing that there is no systematic coarse-scale topographical bias (Tuned normalization: one-way ANOVA slope estimates; $F(2,14) = 0.05$, $p = 0.948$, $\eta_p^2 = 0.009$; post-hoc two-sided one sample t-tests V1: $t(4) = 0.00$, $p = 1$, $d = 0.00$, V2: $t(4) = -2.51$, $p = 0.199$, $d = -1.12$, and V3: $t(4) = -2.49$, $p = 0.202$, $d = -1.11$, Bonferroni corrected; Attention modulation: one-way ANOVA slope estimates; $F(2,14) = 1.16$, $p = 0.239$, $\eta_p^2 = 0.212$; post-hoc two-sided one sample t-tests V1: $t(4) = -1.80$, $p = 0.439$, $d = -0.80$, V2: $t(4) = -2.81$, $p = 0.145$, $d = -1.26$, and V3: $t(4) = -2.49$, $p = 0.831$, $d = -0.56$, Bonferroni corrected). In contrast with previous work, we do not find an increase in attention modulation with eccentricity[1]. This is likely due to differences in stimulus configuration, here participants attended a full field stimulus, which might explain why the magnitude of attention is consistent across eccentricity. Error bars denote ±1 S.E.M. across observers.

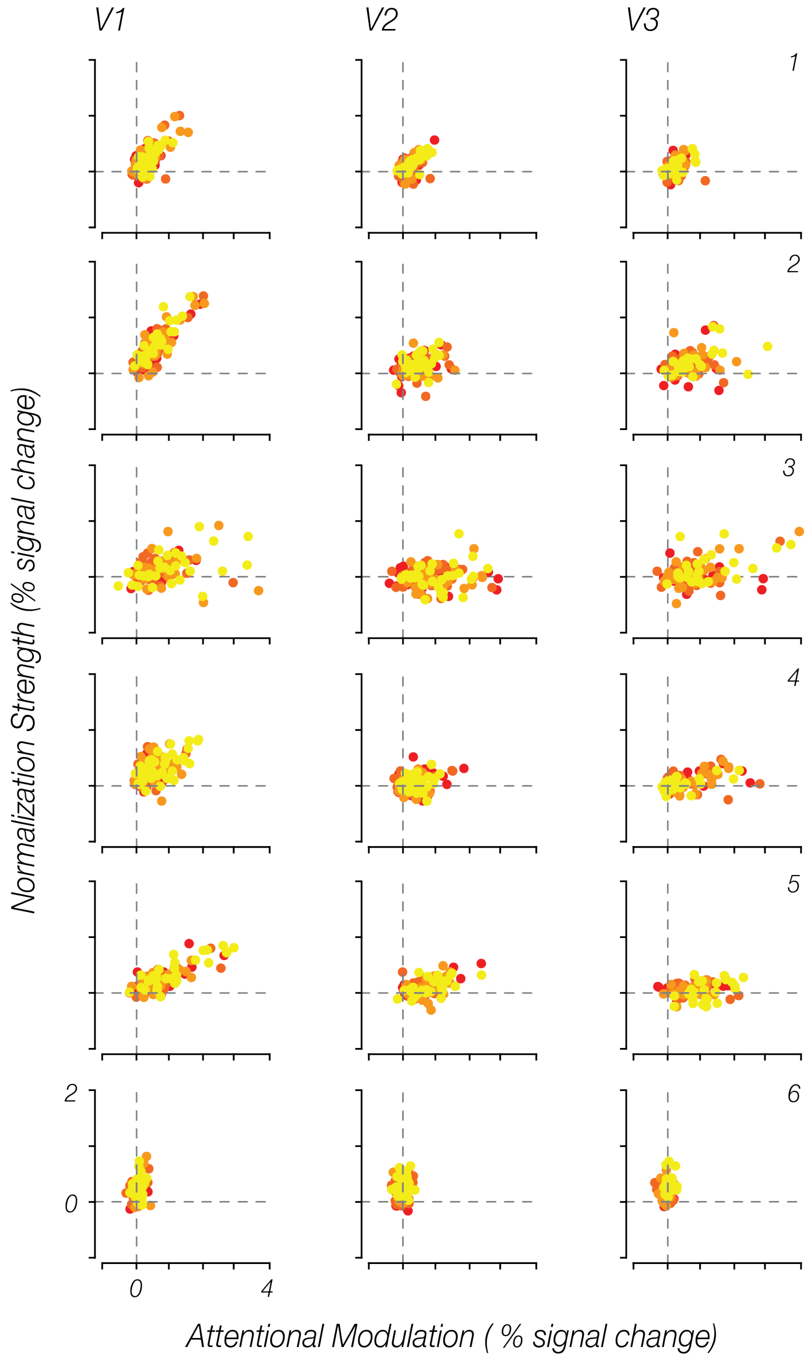

Supplementary Figure 8. Individual participant data illustrating the relationship between attentional modulation and tuned normalization strength. A tight relationship between tuned normalization strength and attentional modulation is evident for the top 25% selected voxels for each observer. Dots illustrate individual voxels within an area, colors represent individual voxels broken up into four bins based on the independent localizer significance (red: bottom 25%, yellow: top 25%); N = 6.

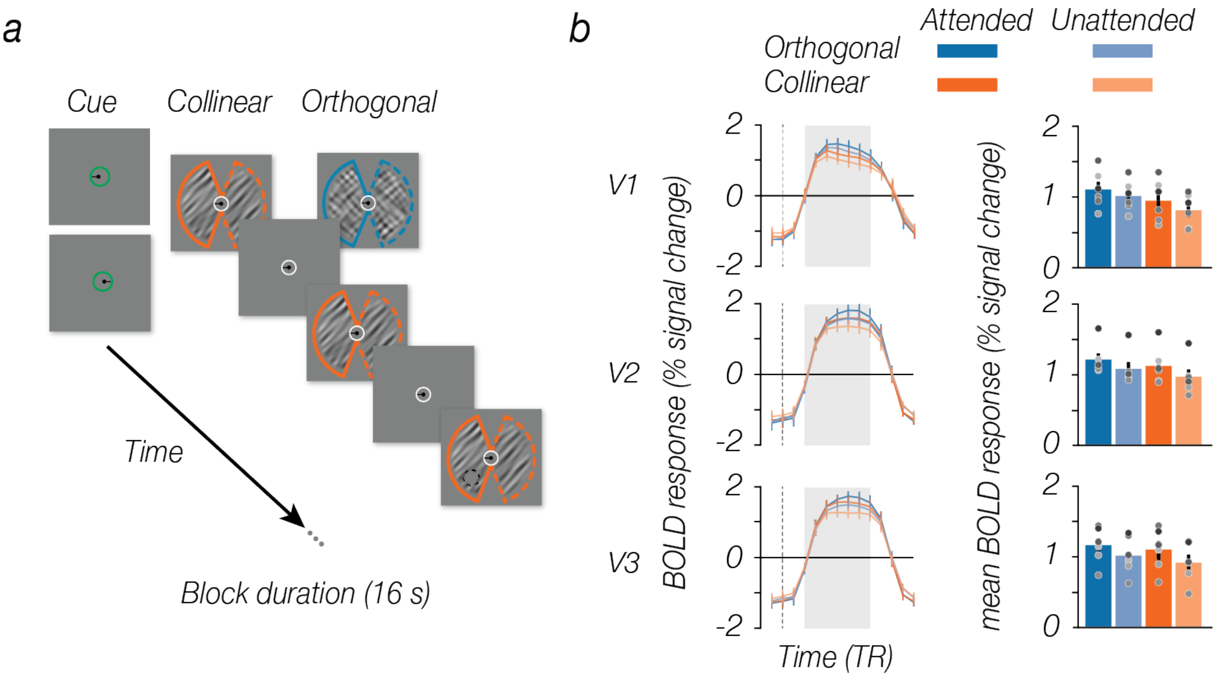

Supplementary Figure 9. Spatial attention modulation for different states of normalization. **a.** Schematic of an example block sequence. Observers were cued to attend either the left or right side of fixation throughout a block (2 sec), after which either collinear (45°/45° or 135°/135°) or orthogonal (45°/135° or 135°/45°) stimuli were presented (250 ms on, 250 ms off). Their task was to detect and discriminate whether a target probe (dashed black circle) appeared anywhere within either the lower or upper visual field of the attended stimulus. Dashed orange/blue lines indicate the unattended side, while the solid lines represent the attended side. Attended side and stimulus configuration conditions were counter-balanced and pseudo-randomized throughout a run. **b.** Mean BOLD responses (left panels) for collinear and orthogonal stimulus configurations with and without covert spatial attention. Grey highlighted part of the BOLD response reflects the section that contributed to the average BOLD response for each participant (right panels). Stimuli are modified for illustrative purposes; dots indicate represent individual participants; N = 6; error bars denote ±1 S.E.M..

References

1. Bressler, D. W., Fortenbaugh, F. C., Robertson, L. C. & Silver, M. A. Visual spatial attention enhances the amplitude of positive and negative fMRI responses to visual stimulation in an eccentricity-dependent manner. *Vision Res* **85,** 104–112 (2013).
