# Supplementary material for: Normalization governs attentional modulation within human visual cortex
Source: Nat Commun. 2019 Dec 11;10:5660. doi: 10.1038/s41467-019-13597-1 (PMC6906520; doi:10.1038/s41467-019-13597-1)
Supplement: Supplementary file 3 — Reporting Summary [file 41467_2019_13597_MOESM3_ESM.pdf]

nature research

Corresponding author(s): Ilona M Bloem

Last updated by author(s): Oct 27, 2019

Reporting Summary

Nature Research wishes to improve the reproducibility of the work that we publish. This form provides structure for consistency and transparency in reporting. For further information on Nature Research policies, see Authors & Referees and the Editorial Policy Checklist .

Statistics

For all statistical analyses, confirm that the following items are present in the figure legend, table legend, main text, or Methods section.

n/a	Confirmed	
☐	☒	The exact sample size (n) for each experimental group/condition, given as a discrete number and unit of measurement
☐	☒	A statement on whether measurements were taken from distinct samples or whether the same sample was measured repeatedly
☐	☒	The statistical test(s) used AND whether they are one- or two-sided<br>*Only common tests should be described solely by name; describe more complex techniques in the Methods section.*
☒	☐	A description of all covariates tested
☐	☒	A description of any assumptions or corrections, such as tests of normality and adjustment for multiple comparisons
☐	☒	A full description of the statistical parameters including central tendency (e.g. means) or other basic estimates (e.g. regression coefficient) AND variation (e.g. standard deviation) or associated estimates of uncertainty (e.g. confidence intervals)
☐	☒	For null hypothesis testing, the test statistic (e.g. F, t, r) with confidence intervals, effect sizes, degrees of freedom and P value noted<br>*Give P values as exact values whenever suitable.*
☒	☐	For Bayesian analysis, information on the choice of priors and Markov chain Monte Carlo settings
☒	☐	For hierarchical and complex designs, identification of the appropriate level for tests and full reporting of outcomes
☐	☒	Estimates of effect sizes (e.g. Cohen's d, Pearson's r), indicating how they were calculated

Our web collection on statistics for biologists contains articles on many of the points above.

Software and code

Policy information about availability of computer code

Data collection	MRI data were acquired at Harvard University's Center for Brain Science Neuroimaging Center (Cambridge, Massachusetts). Data for the first two experiments were collected in a single scan session, using a 3.0 Tesla Tim Trio MRI Scanner. The final experiment was collected using a 3.0 Tesla Prisma MRI Scanner equipped with a 64-channel head coil. Stimuli were generated using Matlab (R2013a) in conjunction with the Psychophysics Toolbox, rendered on a Macbook Pro (OS X 10.7), and were displayed on a rear-projection screen (subtending ~21°x16°) using a gamma-corrected projector. Participants viewed the display through a front surface mirror.
Data analysis	All fMRI analyses were performed in the native space for each participant. Functional volumes were aligned to reconstructed anatomical data, using a surface-based registration between the structural and functional MRI volumes implemented in Freesurfer. Functional data were preprocessed using standard motion-correction procedures, Siemens slice timing correction, and boundary-based registration. To optimize voxel-wise analyses, no volumetric spatial smoothing was performed. Robust rigid registration was performed to align experimental data within each scan session, using the middle time-point of each scan. All further analyses to BOLD responses and behavioral data were conducted using custom code written in Matlab (R2017a).

For manuscripts utilizing custom algorithms or software that are central to the research but not yet described in published literature, software must be made available to editors/reviewers. We strongly encourage code deposition in a community repository (e.g. GitHub). See the Nature Research guidelines for submitting code & software for further information.

Data

Policy information about availability of data

All manuscripts must include a data availability statement. This statement should provide the following information, where applicable:

- Accession codes, unique identifiers, or web links for publicly available datasets
- A list of figures that have associated raw data
- A description of any restrictions on data availability

We have uploaded all preprocessed fMRI and behavioral data, to the Open Science Framework (OSF) at https://osf.io/4qz37. Data is provided to reproduce all Figures in the paper, and Supplementary Information.

Field-specific reporting

Please select the one below that is the best fit for your research. If you are not sure, read the appropriate sections before making your selection.

☒ Life sciences ☐ Behavioural & social sciences ☐ Ecological, evolutionary & environmental sciences

For a reference copy of the document with all sections, see nature.com/documents/nr-reporting-summary-flat.pdf

Life sciences study design

All studies must disclose on these points even when the disclosure is negative.

Sample size	Six healthy adults participated in the first two experiments (3 male, mean age = 30), and seven adults (2 male, mean age = 28) participated in the third experiment. Five adults participated in all three experiments. A power analysis indicated that six subjects would be sufficient to detect the predicted attention effects.
Data exclusions	One observer who participated in the final experiment was excluded from further data analysis, based on consistent eye-movements towards the cued spatial locations (eye-movement analysis revealed a mean deviation from fixation of >1°). No other data was excluded.
Replication	We replicated the main finding of Experiment 1 in Experiment 3 with a slightly different experimental design. This showed that voxels within early visual cortex exhibit tuned normalization.
Randomization	N/A - There were no experimental groups. The order of conditions within an experiment were randomized across participants.
Blinding	Neither data collection nor analysis were performed blind to the conditions of the experiment.

Reporting for specific materials, systems and methods

We require information from authors about some types of materials, experimental systems and methods used in many studies. Here, indicate whether each material, system or method listed is relevant to your study. If you are not sure if a list item applies to your research, read the appropriate section before selecting a response.

Materials & experimental systems

n/a	Involved in the study	
☒	☐	Antibodies
☒	☐	Eukaryotic cell lines
☒	☐	Palaeontology
☒	☐	Animals and other organisms
☐	☒	Human research participants
☒	☐	Clinical data

Methods

n/a	Involved in the study	
☒	☐	ChIP-seq
☒	☐	Flow cytometry
☐	☒	MRI-based neuroimaging

Human research participants

Policy information about studies involving human research participants

Population characteristics	Data was collected from a total of 9 healthy participants. See above.
Recruitment	Participants were recruited from the greater Boston area. All participants were screened for MRI safety, and whether they had normal or corrected-to-normal vision.
Ethics oversight	Boston University Institutional Review board approved this study.

Note that full information on the approval of the study protocol must also be provided in the manuscript.

Magnetic resonance imaging

Experimental design

Design type	Task data, blocked fMRI design
Design specifications	Participants completed 5-10 task runs in Experiment 1 & 2, 8-14 runs in Experiment 3.<br>A run duration was 272s, divided into 16s blocks. Each stimulus block (8 per run) consisted of a 2s cue followed by 14s stimulus presentation. Each run consisted of 8 stimulus blocks (16s duration) interleaved with fixation blocks of equal duration.<br>Additionally, each scan session included 2 functional localizer runs (6 stimulus blocks per run, 16s on, 16s off)
Behavioral performance measures	Experiment 1:<br>Throughout the experiment observers performed a demanding fixation task, finding targets in a rapid letter stream presented at fixation (5Hz, letter size: 0.7°). During stimulus presentation blocks, target letters would appear with a probability of 30%, and participants reported whenever they detected a 'J' or a 'K' amongst distractor letters.<br>Experiment 2:<br>Participants were informed at the start of each stimulus presentation block with a cue (2s) whether to either attend towards the stimuli, or to attend away from the grating (Supplementary Figure 6). During attended stimulus blocks, observers performed an orientation discrimination task, detecting and discriminating a change in the orientation of the stimulus compared to the global orientation (45° or 135°), target stimuli appeared with a probability of 60% throughout the stimulus block. To match task difficulty for the orientation task across observers, we titrated individual thresholds to yield an accuracy of 75%. During unattended stimulus blocks observers performed the same fixation task as described above; target letters appeared with a probability of 30%. All stimulus presentation blocks were completely identical, as both orientation and target letters would appear throughout a block, and only the initial cue informed the participant which task to perform.<br>Experiment 3:<br>A cue (2 s) at the start of each block informed the participant to allocate their covert spatial attention to either the left or right side of a central fixation point, and remained displayed throughout the block (16s total block duration; Supplementary Figure 9). Observers performed a demanding probe detection task, detecting and discriminating whether a neutral gray Gaussian disk appeared at a random location within the upper or lower visual field on the attended side of fixation (probe size 1.5°, with smoothed edges). Probes could appear on either side of fixation throughout a stimulus block.

Acquisition

Imaging type(s)	Functional, structural
Field strength	3 Tesla
Sequence & imaging parameters	all MRI data were acquired at Harvard University's Center for Brain Science Neuroimaging Center (Cambridge, Massachusetts). Data for the first two experiments were collected in a single scan session, using a 3.0 Tesla Tim Trio MRI Scanner (Siemens, Erlangen, Germany) equipped with a 32-channel head coil. A scan lasted 2h, during which we acquired: an anatomical scan (voxel size: 1.2 mm isotropic) using a T1-weighted multi-echo MPRAGE sequence, and functional volumes with whole brain coverage using a simultaneous multislice (SMS) acquisition protocol (69 slices, TR = 2s, TE = 30ms, flip angle = 80°, FoV = 216mm, voxel size = 2mm isotropic, in-plane acceleration factor 3, multiband factor 375,76. The final experiment was collected using a 3.0 Tesla Prisma MRI Scanner equipped with a 64-channel head coil. A scan lasted 1.5-2h, during which we acquired: an anatomical scan (voxel size: 1.2 mm isotropic) using a T1-weighted multi-echo MPRAGE sequence, and functional volumes with whole brain coverage using a SMS acquisition protocol (72 slices, TR = 2s, TE = 30ms, flip angle = 80°, FoV = 208mm, voxel size = 2mm isotropic, in-plane acceleration factor 3, multiband factor 3
Area of acquisition	Whole brain coverage

Diffusion MRI ☐ Used ☒ Not used

Preprocessing

Preprocessing software	Freesurfer 5.3.0 (Fischl, 2012).<br>Functional data were preprocessed using standard motion-correction procedures, Siemens slice timing correction, and boundary-based registration. To optimize voxel-wise analyses, no volumetric spatial smoothing was performed. Robust rigid registration was performed to align experimental data within each scan session, using the middle time-point of each scan.
Normalization	All analysis were performed in the native space for each participant.
Normalization template	The data were not normalized to a template.
Noise and artifact removal	The data was high-passed filtered and a linear regressor was used to account for drift within each run.
Volume censoring	We did not censor volumes.

Statistical modeling & inference

Model type and settings	Task data for all experiments were analyzed by obtaining the activity pattern for each stimulus block, and temporally averaging the BOLD activity across all blocks of the same condition for every voxel within the ROI, after time shifting by 3 TRs to account for the hemodynamic lag.
Effect(s) tested	fMRI data:<br>Repeated-measures ANOVAs and paired-samples t-tests were used to compare the mean BOLD response for each experimental condition. To compare the degree of voxel-wise dependency between attentional modulation and tuned normalization we computed a Spearman correlation, which was Fisher-Z transformed to allow for comparison between observers.<br>Simulation data:<br>To assess the image statistics of our two stimuli configurations we first analyzed the power of the two image classes in the frequency domain using a standard 2-D Fourier transform. We generated 1000 unique bandpass filtered noise images, which were combined either in a collinear or orthogonal configuration, resulting in 500 overlaid stimuli within each image class. Stimuli were fed through a Energy detector model. A bootstrap analysis allowed us to compare whether the two simulated stimulus energy distributions differed from one another by computing the 95% confidence interval.

Specify type of analysis: ☐ Whole brain ☒ ROI-based ☐ Both

Anatomical location(s)	Early visual cortical regions were defined using population receptive field mapping and convential retinotopy methods.
Statistic type for inference (See Eklund et al. 2016)	All statistical tests were performed on the subject level, not individual voxels.
Correction	We applied Bonferroni correction to account for multiple comparisons between different ROIs.

Models & analysis

n/a	Involved in the study
☒	☐ Functional and/or effective connectivity
☒	☐ Graph analysis
☒	☐ Multivariate modeling or predictive analysis
